# Association between extracellular volume control and survival in patients on short daily haemodialysis

**DOI:** 10.1186/s12882-020-01821-w

**Published:** 2020-04-29

**Authors:** Ana Beatriz Lesqueves Barra, Ana Paula Roque-da-Silva, Marcos S. Vasconcellos, Jocemir R. Lugon, Jorge Paulo Strogoff-de-Matos

**Affiliations:** 1 Postgraduation Program in Medical Sciences, Fluminense Federal University (UFF), Niterói, Rio de Janeiro, Brazil; 2 Fresenius Medical Care Brasil, Rio de Janeiro, Brazil; 3 Nephrology Division, Department of Medicine, Fluminense Federal University (UFF), Av. Marques do Parana 303, 2o andar, Niteroi, Rio de Janeiro, 24033-900 Brazil

**Keywords:** Dialysis, Short daily hemodialysis, Bioimpedance, Fluid overload, Survival analysis

## Abstract

**Background:**

Fluid overload (FO) assessed by bioimpedance spectroscopy (BIS) is associated with higher mortality risk in maintenance haemodialysis (HD). The aim was to assess if a better management of FO through short daily haemodialysis (SDHD) could improve survival.

**Methods:**

Retrospective analysis of patients who were on HD 3 sessions/week for at least 3 months and shifted to in-centre SDHD (5 or 6 sessions/week, 2 to 3 h/session) between July 2012 and June 2016 at 23 dialysis units in Brazil. The 12-month risk of death was analysed according to the predialysis hydration status measured before and 6 months after initiation of SDHD. Predialysis hydration status was considered adequate when FO ≤15% of extracellular volume.

**Results:**

A total of 297 patients on SDHD were included in the analysis. Their median age was 57 (IQR 45–67) years, 62% were males, 44% diabetics, 57% on 6 dialysis sessions/week, with a median session duration of 130 (IQR 120–150) minutes. BIS assessment at initiation of the SDHD regimen was performed in 220 patients and FO > 15% was found in 46.4%. Twelve-month survival rates for those with FO ≤15 and > 15% before initiating SDHD were 87.4 and 88.0%, respectively (*P* = 0.92). BIS analysis when completing 6 months on SDHD were available for 229 patients, 26.6% with FO > 15%. The survival rates for the next 12 months (from the 6th to the 18th month of follow-up) for those with FO ≤15 and > 15% were 91.0 and 72.0%, respectively (*P* = 0.0006). In a Cox regression model, after adjustment for demographic, clinical and laboratory variables, FO ≤ 15% persisted associated with a lower mortality risk (hazard ratio 0.34, 95%CI 0.13–0.87).

**Conclusions:**

Moving from conventional HD to SDHD was associated with better control of excessive extracellular volume. Patients who reached or maintained predialysis fluid overload ≤15% after initiating SDHD presented a lower risk of death.

## Background

Mortality in maintenance haemodialysis (HD) patients is still unacceptably high, and cardiovascular disease continues to be the leading cause of death and hospitalizations in that population [1]. Although multiple factors inherent to renal disease are responsible for this risk, the intermittent nature of the conventional HD schedules may have an important contribution [2]. The establishment of HD prescription of a 3 to 4 h sessions, 3 times a week was not based on clinical outcomes. Such dialysis schedule was conceived in the 1970s to face economical and logistic challenges and devised to accommodate a greater number of patients with end-stage renal disease [3].

The standard HD many times does not fit the individual patient’s needs, especially for appropriate fluid management. Short daily haemodialysis (SDHD) can improve extra-cellular volume management leading to a better blood pressure control and reduction of left ventricular mass as compared to the 3-session per week HD regimen [4]. However, the estimation of HD patient dry weight using only clinical parameters is a challenge in clinical practice. Bioimpedance is a useful tool to improve the diagnosis of hydration status and the management of fluid in dialysis patients. Small clinical trials have shown that the use of bioimpedance to guide the volume management improved blood pressure control, decreased left ventricular mass and reduced mortality [5, 6].

Overhydration or fluid overload (FO), defined as the presence of more than 15% of the extracellular water, has been associated with higher mortality risk in patients on conventional HD [7–9]. Nevertheless, the association between fluid overload measured by bioimpedance and the risk of death in SDHD is not yet known. In the present study, we analyse if better management of overhydration through SDHD could have a positive impact on survival.

## Methods

This is a retrospective analysis of adult patients (≥18 years old) from 23 dialysis facilities in Brazil on HD 3 sessions/week for at least three months who were shifted to in-centre SDHD between July 2012 and June 2016. The main indication for the modification of the dialysis schedule was inadequate fluid management on conventional HD diagnosed on clinical grounds. Due to limitations of more frequent HD reimbursement by the public health system, only patients with private health insurances were shifted to that HD regimen.

All demographic, clinical, laboratory and BIS data were extracted from the European Clinical Dialysis Database (EuCliD®), a standardized electronic medical record used by all participating centres. This study was approved by the local ethics committee.

In all dialysis centres, the routine laboratory exams were performed monthly and blood samples were collected before the midweek dialysis session. BUN was also measured post-dialysis. Parameters of dialysis treatment and blood pressure values were defined as the mean of all measurements in the last 4 weeks. Dialysis adequacy was expressed as equilibrated Kt/V for urea [10] and converted to weekly standard Kt/V for patients on SDHD [11].

Body composition and hydration status were evaluated using a portable whole body bioimpedance spectroscopy (BIS) device (BCM® - Body Composition Monitor, Fresenius Medical Care, Bad Homburg, Germany). BIS assessments were always performed immediately before dialysis sessions with the patient sitting in the dialysis chair. BIS assessment was recommended for all patients every 3 months, except in case of contraindication such as the use of metal hip prosthesis. Adequate hydration was defined as a relative FO/extracellular volume below 15%.

The last BIS evaluation before increasing the frequency of treatment was chosen to define the hydration status before SDHD. The last BIS evaluation before completing the 6th month on SDHD was taken to define the hydration status after SDHD.

**Fig. 1:**
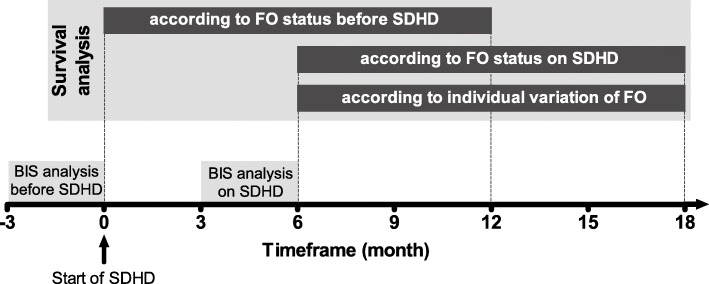
Design of survival analysis according to the FO status before and after starting SDHD. Schematization of the three different survival analysis: according to the fluid overload (FO) status assessed by bioimpedance spectroscopy (BIS) before starting short daily haemodialysis (SDHD); FO status after switching to SDHD; or the individual variation of FO status comparing BIS analysis before and after starting SDHD

Three different 12-month survival analyses were accomplished. First, we looked at survival from month 0 to 12 according to the hydration status (FO ≤15% versus > 15%) before the change to SDHD. Second, we analysed survival from month 6 to 18 but considering the hydration status (FO ≤15% versus > 15%) after at least three months on SDHD. Finally, four survival curves were compared according to the individual variation of the hydration status (Δ FO) before and after moving to SDHD: patients who remained with adequate extracellular volume control (adequate - adequate); patients who were initially overloaded but became normohydrated (high - adequate); patients who were fluid overloaded and so remained after switching to SDHD (high - high); and patients who eventually had a deterioration of fluid control on SDHD (adequate - high), Fig. 1.

### Statistical analysis

Kolmogorov-Smirnov was used to test for the distribution of variables. Variables with normal distribution were expressed as mean ± standard deviation; those with non-Gaussian distribution, as median and interquartile intervals. T-test or Mann-Whitney was used as appropriate when two groups were compared. The Kaplan-Meier method was used for the analysis of survival, and comparison between curves was made by the Log-Rank test. Overhydration as a predictor of death risk was analysed in a multivariate Cox regression model. In all analysis, *P* < 0.05 were considered significant. The software SPSS, version 21.0 (Chicago, Illinois, USA) was used for the statistical analysis.

## Results

**Fig. 2:**
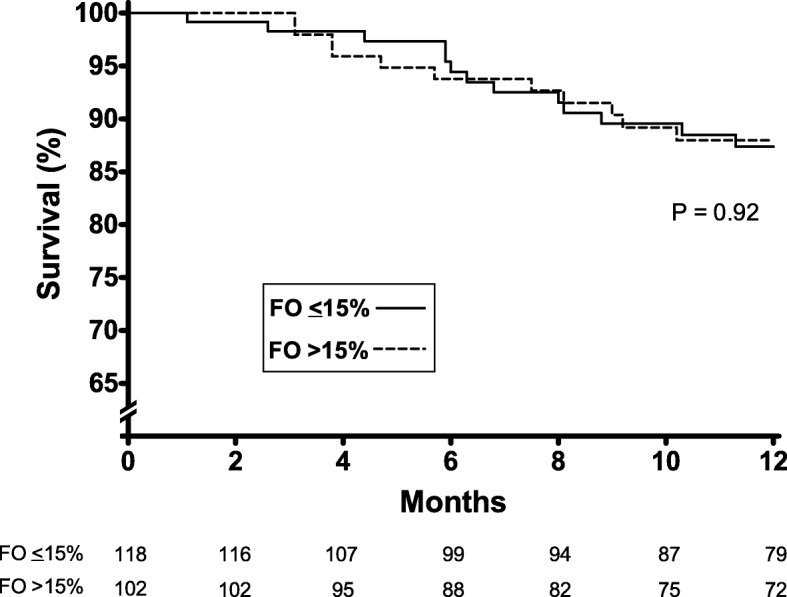
Survival curves on SDHD according to the hydration status before initiating SDHD

A total of 297 patients on SDHD were included in the analysis. The characteristics of the patients are presented in Table 1. BIS assessment before initiating SDHD was performed in 220 patients and 102 (46.4%) of them had initial FO > 15%. Patients with FO > 15% before initiating SDHD was older, had a higher prevalence of diabetes and central intravenous catheter as the vascular access, and lower levels of serum albumin than the ones with FO ≤15% (Table 1). The survival rates for the first 12-month on SDHD were 87.4% for those with FO ≤15 and 88.0% for those with FO > 15% (*P* = 0.92), Fig. 2.

**Table 1:** Profile of patients whereas on conventional haemodialysis immediately before shifting to short daily haemodialysis

Variables	All patients	FO ≤15%	FO > 15%	*P value*
	(*n* = 297)	(*n* = 118)	(*n* = 102)	
FO (%ECV)^§^	13.8 (5.1–22.1)	6.2 (1.9–10.4)	23.9 (17.8–30.0)	–
Age (years)	57 (45–67)	53 (39–63)	61 (48–70)	*< 0.0001*
Male gender, n (%)	189 (63.6)	70 (59.3)	70 (68.6)	*0.16*
Diabetes, n (%)	128 (43.1)	43 (36.4)	52 (51.0)	*0.04*
Charlson comorbidity index	4 (2-4)	3 (2-4)	4 (2–4)	*0.018*
Vintage (months)	29 (13-61)	33 (13-61)	29 (16-70)	*0.38*
HBsAg+, n (%)	3 (1.0)	1 (0.8)	1 (1.0)	*1.00*
Anti-HCV+, n (%)	9 (3.0)	4 (3.4)	3 (3.0)	*1.00*
Anti-HIV+, n (%)	6 (2.0)	4 (3.4)	1 (1.0)	*0.39*
Vascular access, n (%)				
Native AFV	210 (70.7)	97 (82.2)	69 (67.6)	*0.018*
Graft	13 (4.4)	6 (5.1)	2 (2.0)	*0.29*
Catheter	74 (24.9)	15 (12.7)	31 (30.4)	*0.002*
Treatment time (min/session)	240 ± 10	239 ± 10	240 ± 10	*0.36*
Equilibrated KT/V	1.28 ± 0.35	1.29 ± 0.37	1.27 ± 0.33	*0.81*
Body mass index (Kg/m^2^)	25.5 ± 5.6	26.8 ± 6.2	24.0 ± 5.0	*< 0.0001*
Body weight (Kg)	71.0 ± 17.3	74.8 ± 18.8	67.3 ± 15.1	*0.0003*
UF rate (mL/hour/Kg)	9.9 ± 3.5	9.9 ± 3.4	10.1 ± 3.6	*0.71*
Predialysis SBP (mmHg)	139 ± 21	138 ± 19	141 ± 22	*0.19*
Predialysis DBP (mmHg)	76 ± 13	77 ± 12	74 ± 14	*0.018*
Postdialysis SBP (mmHg)	134 ± 20	132 ± 18	138 ± 22	*0.008*
Postdialysis DBP (mmHg)	75 ± 12	76 ± 12	74 ± 13	*0.12*
Haemoglobin (g/dL)	10.5 ± 1.9	10.9 ± 1.8	10.1 ± 1.9	*0.002*
Serum albumin (g/L)	37.7 ± 4.8	39.1 ± 4.0	37.0 ± 5.2	*0.0002*
Phosphorus (mg/dL)	5.1 ± 1.6	5.3 ± 1.5	5.1 ± 1.7	*0.24*

Data are expressed as frequency (%), mean ± standard deviation or median (interquartile interval)

*FO* fluid overload; *ECV* extracellular volume; *AVF* arteriovenous fistula; *UF* ultrafiltration; *SBP* systolic blood pressure; *DBP* diastolic blood pressure; ^§^ Data available in only 220 patients

After 6 months, 256 patients remained on SDHD. By that time, the mean blood pressure levels were reduced, and the mean serum albumin, bicarbonate and haemoglobin were higher with no significant change in serum phosphate (Table 2).

**Table 2:** Changes in body composition by BIS assessment and selected laboratory parameters from the first to the sixth month on short daily haemodialysis

Variables	Month 0	Month 6	*P value*
	(*n* = 297)	(*n* = 256)	
FO (%ECV)^§^	13.8 (5. 1-22.1)	7.6 (− 0. 4-15.3)	*< 0.0001*
Fat mass (%)^§^	36 (27–43)	38 (29–44)	*0.13*
Lean mass (%)^§^	47 (39–59)	46 (37–56)	*0.38*
Dry weight (Kg)	71.0 ± 17.3	71.2 ± 16.6	*0.91*
Predialysis SBP (mmHg)	139 ± 21	133 ± 21	*0.0011*
Predialysis DBP (mmHg)	76 ± 13	72 ± 12	*0.0004*
Postdialysis SBP (mmHg)	134 ± 20	129 ± 20	*0.0018*
Postdialysis DBP (mmHg)	75 ± 12	72 ± 12	*0.0016*
Haemoglobin (g/dL)	10.5 ± 1.9	10.9 ± 2.0	*0.050*
Serum albumin (g/L)	37.7 ± 4.8	39.6 ± 4.1	*< 0.0001*
Predialysis BUN (mg/dL)	57.3 ± 19.4	53.6 ± 17.9	*0.035*
Phosphorus (mg/dL)	5.1 ± 1.6	5.1 ± 1.5	*0.24*
Calcium (mg/dL)	9.1 ± 0.8	9.1 ± 0.8	*0.76*
Potassium (mEq/L)	5.2 ± 0.9	5.1 ± 0.8	*0.19*
Sodium (mEq/L)	138 ± 4	137 ± 5	*0.29*
HCO3- (mEq/L)	20.9 ± 2.9	22.4 ± 3.0	*0.031*

Data are expressed as mean ± standard deviation or median (interquartile interval)

*FO* fluid overload; *ECV* extracellular volume; *BUN* blood urea nitrogen; *SBP* systolic blood pressure; *DBP* diastolic blood pressure; § Data available in only 220 patients at month 0 and 229 patients at month 6

Data from BIS analysis before completing 6 months on SDHD were available for 229 out of 256 patients. Median FO was reduced from 13.8% (interquartile range [IQR] 5.1–22.1%) to 7.6% (IQR -0.4 – 15.3%), *P* < 0.0001 (Table 2) and the rate of patients with FO > 15% dropped from 46.4 to 26.6% (*P* = 0.0005).

### Hydration status on SDHD and survival

**Fig. 3:**
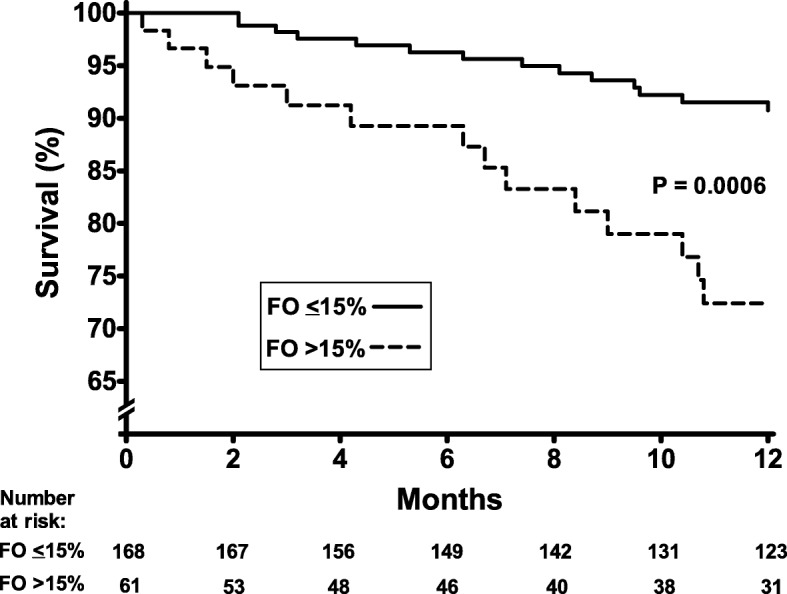
Survival curves on SDHD according to the hydration status after switching to SDHD. Survival curves from 6th to 18th month on short daily haemodialysis (SDHD) according to the hydration status after switching to SDHD

The survival rates from the 6th to the 18th month on SDHD, for those with FO ≤15% or > 15% before the start of this timeframe were 91 and 72%, respectively (*P* = 0.0006), Fig. 3. Patients with FO > 15% on SDHD tended to be older, with a predominance of the male gender. They also presented a lower body mass index, serum albumin and haemoglobin (Table 3).

**Table 3:** Characteristics of patients at the 6th month on short daily haemodialysis according to the hydration status

Variables	FO ≤15%	FO > 15%	*P value*
	(*n* = 168)	(*n* = 61)	
FO (%ECV)	3.5 (−2. 4-9.0)	22.3 (17. 9-27.1)	–
Age (years)	55 (41–66)	61 (47–73)	*0.086*
Male gender (%)	57.7	70.5	*0.092*
Diabetes (%)	37.5	49.2	*0.13*
Charlson comorbidity index	3 (2-4)	4 (2-4)	*0.066*
Vintage (months)	28 (11-60)	39 (17-70)	*0.13*
Vascular access (%)			
Native AFV	74.4	62.3	*0.098*
Graft	3.0	8.2	*0.14*
Catheter	22.6	29.5	*0.30*
HD frequency (%)			
5 sessions/week	41.1	55.7	*0.052*
6 sessions/week	58.9	44.3	
Treatment time (min/week)	814 ± 189	779 ± 139	*0.19*
Standard KT/V	3.45 ± 1.01	3.15 ± 0.85	*0.067*
Body mass index (Kg/m^2^)	26.1 ± 5.8	23.6 ± 5.4	*0.003*
Dry weight (Kg)	72.0 ± 16.4	68.9 ± 17.1	*0.21*
UF rate (mL/hour/Kg)	10.8 ± 3.2	10.5 ± 3.8	*0.58*
Pre-HD SBP (mmHg)	133 ± 21	133 ± 21	*0.96*
Pre-HD DBP (mmHg)	73 ± 11	69 ± 14	*0.053*
Post-HD SBP (mmHg)	128 ± 19	133 ± 22	*0.086*
Post-HD DBP (mmHg)	72 ± 11	70 ± 14	*0.18*
Haemoglobin (g/dL)	11.2 ± 2.0	10.1 ± 1.8	*0.0002*
Serum albumin (g/L)	40.0 ± 3.7	38.3 ± 4.6	*0.004*
Phosphorus (mg/dL)	5.3 ± 1.5	4.8 ± 1.3	*0.062*

Data are expressed as mean ± standard deviation or median (interquartile interval)

FO, fluid overload; ECV, extracellular volume; AVF, arteriovenous fistula; UF, ultrafiltration; SBP, systolic blood pressure; DBP, diastolic blood pressure

In a Cox regression model, the crude mortality risk was reduced for patients with FO ≤15% (hazard ratio 0.30, 95%CI 0.14–0.62). After adjustment for demographic, clinical and laboratory variables, FO ≤15% on SDHD persisted associated with lower mortality risk (hazard ratio 0.34, 95%CI 0.13–0.87). In the same analysis, older age, low serum albumin and predialysis systolic blood pressure below 130 mmHg were associated with higher mortality risk (Table 4).

**Table 4:** Cox regression models for prediction of one-year death risk in 229 patients with bioimpedance assessment at the 6th month on short daily haemodialysis by levels of adjustment

Variables	Model 1	Model 2	Model 3
	Hazard ratio (95% confidence interval)		
FO ≤15%	0.30 (0.14–0.62)	0.26 (0.11–0.61)	0.34 (0.13–0.87)
Age (year)	–	1.05 (1.02–1.09)	1.05 (1.02–1.08)
Male gender	–	0.82 (0.36–1.88)	1.0 (0.39–2.55)
Diabetes	–	1.30 (0.55–3.05)	1.47 (0.59–3.68)
Vintage (month)	–	1.00 (0.99–1.01)	1.00 (0.99–1.01)
Catheter	–	1.49 (0.59–3.75)	1.49 (0.59–3.75)
BMI (Kg/m^2^)	–	1.06 (0.98–1.15)	1.09 (0.99–1.19)
SBP < 130 mmHg	–	2.06 (0.93–4.59)	2.46 (1.06–5.69)
UF rate (mL/h/Kg)	–	1.06 (0.92–1.23)	1.07 (0.92–1.25)
Standard KT/V	–	–	1.01 (0.47–2.16)
Serum albumin (g/L)	–	–	0.88 (0.79–0.99)
Phosphorus (mg/dL)	–	–	0.79 (0.56–1.10)
Haemoglobin (g/dL)	–	–	0.95 (0.76–1.19)

*Model 1: unadjusted hazard ratio, Model 2: adjustment for clinical and demographics; Model 3: model 2 plus laboratory variables. FO, fluid overload; BMI, body mass index; SBP, systolic blood pressure; UF, ultrafiltration*

### Change in hydration status after shifting to SDHD

One hundred and seventy-eight had both BIS assessments (before and after initiating SDHD) allowing evaluation of their change in the hydration status. Taking the hydration status of the two studied periods, patients were distributed in following categories: 81 (46%) patients were in the adequate - adequate; 13 (7%) in the adequate - high; 38 (21%) in the high - high; and 46 (26%) in the high - adequate.

**Fig. 4:**
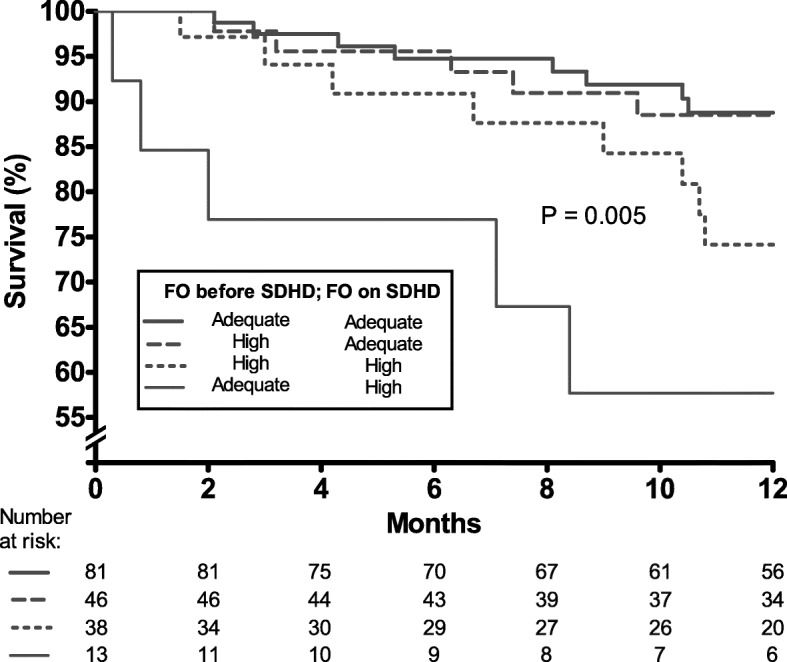
Survival curves on SDHD according to the hydration status before and after switching to SDHD. Survival curves from 6th to 18th month on short daily haemodialysis (SDHD) according to the combination of hydration status in two different occasions (before and after switching to SDHD)

The survival rate from the 6th to 18th month on SDHD was 89% for patients with adequate hydration status on SDHD regardless of their hydration status on conventional HD, Fig. 4. On the other hand, for patients who either persisted with a high hydration status or switched from an initially adequate hydration status to a worse control after initiation of SDHD, the survival rate was poorer (74 and 58%, respectively).

## Discussion

Our findings suggest that SDHD can improve volume control in many patients who were not previously well controlled in the conventional HD regimen of 3 sessions per week. Maintaining or reaching a hydration status up to 15% above the normal volume, as estimated for the extracellular fluid measured by bioimpedance, was associated with a significant reduction in the risk of death in the following 12 months. Such finding is suggestive that the same cut-off point, used in previous studies with patients on conventional HD, could also be applied for patients on SDHD [7, 8]. Recently, Zoccali et al. [9] analysed the association between hydration status and death risk in almost 40 thousand HD patients, with more 200,000 BIS evaluations. Overhydration was strongly associated with the risk of death. In that study, inadequate hydration status was defined as FO > 15% for men and > 13% for women.

Wizemann et al. [7] found a more than 2-fold increase in the risk of death in 269 patients on conventional HD who were fluid overloaded at the beginning of a 3. 5-year follow-up period. In that study, only 22% of the patients were overloaded (FO > 15%), a number that contrasts with our sample in which FO > 15% was found in 46% of patients before moving to SDHD. Shifting to SDHD regimen could be seen as a strategy to improve patients’ hydration status [4]. Indeed, only 26.6% of our patients were still fluid overloaded after 6 months on SDHD. As the majority of them had been on conventional HD for more than a year before moving to SDHD, with a median of 29 months on renal replacement therapy, those patients probably would not have had any improvement in their hydration status if they had been maintained in the same regimen of HD 3 times per week.

The intermittent nature of the in-centre conventional HD schedules could have a relevant role in the high mortality rate in the dialysis population. Two factors related to volume overload may contribute to a worse prognosis: the higher left ventricular preload related to the long periods of fluid accumulation, favouring left ventricular hypertrophy, and the excessive ultrafiltration rate [12, 13]. Left ventricular hypertrophy is associated with high all-cause and cardiovascular mortality in dialysis patients and is usually used as a surrogate marker of a dismal course in dialysis. In the Frequent Hemodialysis Network Daily Trial, SDHD led to a significant reduction of left ventricular mass [4].

In the present study, switching to a more frequent treatment also impacted positively on blood pressure control, serum bicarbonate, albumin and haemoglobin. On the other hand, no improvement in the serum phosphorus control was seen and only a modest reduction in predialysis BUN was found 6 months after starting SDHD. We hypothesize that this could be a consequence of improved appetite and, consequently, increased protein intake.

For decades, the concept of dry weight in HD has been accepted as the weight that keeps patient normotensive without the need of hypotensive drugs. In fact, appropriate management of imbalanced fluid status can adequately control blood pressure in many cases [14]. However, determining dry weight based on trial and error is particularly challenging to hypotensive patients who are simultaneously hypervolemic. In this study, we found that predialysis systolic blood pressure below 130 mmHg was independently associated with a more than 2-fold increase in the risk of death. As seen by Zoccali et al. [9], the risk of death was higher in the subgroup of patients who presented overhydration concomitant to predialysis systolic blood pressure below 130 mmHg, compared to the group without overhydration and predialysis systolic blood pressure between 130 and 160 mmHg.

Just recently, complementary methods for assessing volume status, such as BIS and lung ultrasound, have turned the search for dry weight less subjective. We found that even after moving to a more frequent treatment and having BIS as an accessory tool, many patients persisted with excessive overhydration and had reduced survival rate. It is hard to determine whether this reflects unfavourable clinical conditions or simply if the overhydration condition was overlooked and the patients were not properly managed after moving to SDHD. In the latter hypothesis, the opportunity for appropriate intervention in a modifiable variable with the potential to improve the prognosis could have been missed.

The deterioration of hydration status after moving to SDHD was only seen in 13 patients. They had adequate hydration control on conventional HD but unexpectedly deteriorated their hydration status after moving to SDHD. That subgroup of patients had a very poor outcome, with only 58% survival rate at 12 months. It is possible that the worsening of those patients could be a consequence of a decline in their clinical condition, from causes not related to the treatment, leading to a weight loss not promptly recognized and without the due adjustment in their dry weight.

Another method to diagnose overhydration in haemodialysis patients is the B-lines score through the lung ultrasound. Zoccali et al. [15] found moderate-to-severe lung congestion in 45% and very severe congestion in 14% of patients on HD, and the vast majority of them was asymptomatic or presented slight symptoms of heart failure. Those patients with very severe congestion had more than 4-fold risk of death than the patients having mild or no congestion. There is an ongoing clinical trial designed to evaluate the effects of dry weight adjustment guided by lung ultrasound on clinical outcomes [16]. Interim results showed an effective and safe reduction of ambulatory blood pressure levels [17]. A limitation of lung ultrasound in the daily clinical practice is the dependence of a trained doctor for its execution [18]. Thus, compared to lung ultrasound, BIS is an easy-to-perform tool which can be used as many times as indicated.

In-centre SDHD is a well-established modality of therapy in the literature, which is associated with improved health related quality of life as compared to conventional haemodialysis [19], but it does require that patients have to attend the dialysis centre more frequently. In this regard, there may be room for home haemodialysis that could offer the benefits reported in the present study with more autonomy and perhaps a better quality of life [20].

Our study presents several limitations, including the retrospective nature of the analysis and missing BIS assessment data for a significant number of patients, both at baseline and the 6th month on SDHD. Other weakness of the study is the lack of uniformity regarding the interdialytic interval preceding the BIS assessment. Patients underwent BIS assessment always immediately before the dialysis session, but there was no control of the day of the week in which it was performed or the interdialytic interval that preceded the data collection. Finally, adjustments could only be performed by confounders that were available in the database, a limitation inherent to retrospective studies. The study also has its strengths, such as the inclusion of a large number of patients and the standardization of procedures, evaluations and register of data in all dialysis facilities.

## Conclusion

Moving from conventional haemodialysis to SDHD was associated with better control of excessive extracellular volume and blood pressure, and improvement in several laboratory parameters. Patients who reached adequate hydration control after initiating SDHD, i.e., up to 15% above the normal volume estimated for the extracellular fluid, presented a lower risk of death. Thus, pursuing an adequate extracellular volume control by intensifying haemodialysis frequency can be a strategy to manage a variable that impacts on survival.

## Data Availability

The datasets used and analysed during the current study are available from the corresponding author on reasonable request.

## Abbreviations

BIS: Bioimpedance spectroscopy
FO: Fluid overload
HD: Haemodialysis
SDHD: Short daily haemodialysis
